# Can Contrast Effects Regulate Emotions? A Follow-Up Study of Vital Loss Decisions

**DOI:** 10.1371/journal.pone.0042763

**Published:** 2012-08-08

**Authors:** Qi Li, Yue Qi, Xianyun Liu, Jing Luo

**Affiliations:** 1 Key Laboratory of Behavioral Science, Institute of Psychology, Chinese Academy of Sciences, Beijing, China; 2 State Key Laboratory of Brain and Cognitive Science, Institute of Psychology, Chinese Academy of Sciences, Beijing, China; 3 Graduate University of the Chinese Academy of Sciences, Beijing, China; 4 Academy of Psychology and Behaviour, Tianjin Normal University, Tianjin, China; 5 Beijing Key Laboratory of Learning and Cognition, Department of Psychology, Capital Normal University, Beijing, China; 6 Key Laboratory of Mental Health, Institute of Psychology, Chinese Academy of Sciences, Beijing, China; University of Medicine & Dentistry of NJ - New Jersey Medical School, United States of America

## Abstract

Although many studies focus on the how contrast effects can impact cognitive evaluations, the question of whether emotions are regulated by such contrast effects is still the subject of considerable debate, especially in the study of loss-related decisions. To address this gap in the literature, we designed three decision making loss conditions: (i) both losses are trivial (TT), (ii) one loss is trivial and the other loss is vital (TV), or (iii) one loss is trivial and the other loss is routine (TR). In study 1, which compared the difference between the negative emotion ratings in TT and TV, we found that negative emotions were affected by the contrast effects. In study 2, which compared the difference between the importance of trivial options in TT and TV, we found that the contrast effects differentially changed the importance of trivial options in the two conditions, which in turn down-regulated negative emotions. In study 3, the impact of decision difficulty was controlled by predetermining the items to be lost. In this study, we found that, when comparing the differences between the negative emotions of losing trivial options in TV and TR, the contrast effects still modulated the loss-related emotions. We concluded that the contrast effects could down-regulate emotions. To our knowledge, this is the first demonstration that contrast effects can alleviate negative affect in loss-related decision making. This study will enrich and extend the literature on emotion regulation theory, and it will provide a new cost-effective mitigation strategy for regulating negative emotions.

## Introduction

Contrast effects have been conceptualized in previous research as the intensification and reduction of cognition as a result of immediately previous or simultaneous exposure to a stimulus of lesser or greater value in the same dimension [1]–[7]. For example, in size perception, the Ebbinghaus illusion is an optical illusion of relative size perception. In the best-known version of the illusion, two target circles of equal size, each of which are surrounded by a circular array of either smaller or larger circles, are presented side by side. Subjects typically report that the target circle surrounded by the array of smaller circles appears larger than the circle surrounded by the array of larger circles [8], [9]. Contrast effects are ubiquitous in cognitive processing. These effects were first demonstrated in the field of psychophysics across a variety of sensory modalities, including judgments of the heaviness of lifted weights, the temperature of water, or the brightness of lights [10]–[12]. Furthermore, contrast effects have been found to affect perception [8], [9], [13] and higher cognitive processes, such as assessment [14] and representation [15].

An analysis of the emotional reactions of bronze and silver medalists at the 1992 Summer Olympics indicated that bronze medalists tended to be happier than silver medalists [16]. When compared with the bronze medalists, why did the silver medalists feel less happy even though they ranked higher? One possible explanation was that their references of judgments were different: for the silver medalists, the reference of judgment was winning the gold, whereas for the bronze medalists, the reference of judgment was finishing without a medal. As the bronze medalists and sliver medalists compared their different references, the formers were happier than the latter. With this concept in mind, can we form the conclusion that contrast effects affect both cognitive processing and emotion regulation?

Our previous imaging research investigated the neural activities of vital-trivial loss decisions (in which one option was vital while another was trivial) versus trivial-trivial loss decisions (in which both options were trivial) and found increased activation in the orbitofrontal cortex (OFC) and the striatum. These two areas were found to be related to reward outcome and positive emotions [17]–[24]. For example, OFC is responsible for calculating the value of a reward outcome [24] and supporting positive emotions related to reward attainment [20], and striatum mediates important aspects of decision-making on the basis of the actions' reward value [17]. However, evidence has also indicated that with increased positive emotion or decreased negative emotion, there is a connection between the two areas, thus, the OFC and striatum were called relief-related areas [19]–[22], [25], [26]. Similar findings have reported that contrast effects could regulate the activation of relief-related areas. For example, previous studies utilizing monetary [21] and shock [19] stimuli suggested that relief-related emotion was experienced when the decision maker contrasted the chosen and unchosen outcomes [19], [27]–[29]. If the unchosen outcome was worse than the chosen outcome, then increased activation was observed in the relief-related areas, such as dorsolateral prefrontal cortex (DLPFC) and striatum, and participants reported decreasing regret for their optimal choices [19], [21]. However, in our study, no significant differences were observed in the negative affect ratings made during decision making between the trivial-trivial and vital-trivial loss conditions [22]. Moreover, Hanselmann et al. [30] examined the effects of two distinct trade-off types on emotions: taboo trade-offs (i.e., a scenario with a sacred value against a secular value) and routine trade-offs (i.e., a scenario with two secular values against each other). After being presented with the final decision situation, the participants were administered the PANAS as a way to measure the positive and negative emotions associated with the decision situation. The results revealed that, although the participants in the taboo trade-off condition tended to feel more negative emotions than those participants in the routine trade-off condition, this difference failed to reach significance.

The aforementioned research demonstrates that contrast effects regulated the activity of the relief-related neural areas shown in the neuroimaging data. However, the contrast effects did not affect the emotions associated with decisions in the subjective negative affect ratings of choice. It is likely that the imaging data were acquired on-line during decision making, whereas the negative affect ratings were retrospectively recollected after each block was finished [22]. Thus, the behavioral measure might be inadequate, because the block design results in the loss of abundant instant emotion information, in which case the emotions aroused from various situations cannot be distinguished. In contrast, an event design that examines responses to individual trials might reflect the current mood in a more timely and effective manner.

To address the limitations of previous studies [22], we conducted a follow-up study using an event-related design to obtain more accurate evidence for how concerning whether contrast effects regulate emotions. We designed three loss conditions that involved decision making: (i) trivial-trivial loss decision (TT), in which the two options are both trivial (e.g., losing a table lamp or losing a telegram); (ii) trivial-vital loss decision (TV), in which one of the two options is trivial while the other option is vital (e.g., losing a table lamp or losing a leg); and (iii) trivial-routine loss decision (TR), in which one of the two options is trivial while the other option is routine (e.g., losing a table lamp or losing rice). In study 1, we compared the difference in negative emotions between TT and TV to examine whether negative emotions were affected by the contrast effects. In study 2, we explored whether contrast effects could differentially change the importance of trivial options in two conditions (TT and TV), which in turn down-regulated negative emotions. In study 3, loss was predetermined to control for the impact of decision difficulty on emotions. As a way to determine whether contrast effects could still affect loss-related emotions, we compared the difference in the negative emotions elicited when losing trivial options between TV and TR.

## Materials and Methods

### Participants

Thirty healthy university students participated in Experiments 1 (12 males, 20.30±.37 years old), 2 (7 males, 22.37±.35 years old) and 3 (13 males, 21.43±.26 years old). All of the participants reported a lack of neurological or psychiatric history. Each participant voluntarily enrolled and signed an informed consent statement prior to the experiment. The procedure was approved by the Institutional Review Board of the Institute of Psychology, Chinese Academy of Sciences.

### Stimuli

Initially, we selected 1,100 high-frequency Chinese nouns consisting of 2 characters each [31]. The relative importance and familiarity of each noun was rated by a separate group of 35 participants on a 7-point scale (1 to 7: unimportant to extremely important; unfamiliar to extremely familiar). Importance was defined as how desirable or valuable the item was to each individual's life. The final stimulus set consisted of four groups of 20 nouns each. The familiarity ratings were constant across all four groups.

For Experiments 1 and 2, the importance rating for one noun group (mean ± SE = 5.92±.06) was significantly higher than the ratings of the other three groups (mean ± SE = 2.72±.12, 2.71±.12, 2.71±.10, *ps*<.001). However, the importance ratings did not differ significantly among the three latter groups. Due to this group difference, the nouns from the four groups were subsequently paired in terms of their importance, thus resulting in two sets of 20 pairs: trivial-trivial (TT) and trivial-vital (TV).

For Experiment 3, the nouns were categorized according to their importance ratings, thus resulting in one “high importance” group (mean ± SE = 6.02±.08), one “medium importance” group (mean ± SE = 4.70±.10) and two “low importance”groups (mean ± SE = 2.62±.16, 2.59±.17). There was a significant difference in the importance ratings between these four groups (*ps*<.001), although no significant differences were found between the two “low importance” groups. Because of these rating results, the nouns from the three categories were subsequently paired in terms of their importance, thus resulting in two sets of 20 pairs: trivial-routine (TR) and trivial-vital (TV).

### Procedure

Prior to the test, the participants completed two specific “loss” examples as a way to maximally involve them in the experimental situation. The first example was considered trivial and involved the “*Loss of a cola.*” In this example, the participants were instructed to drink a small amount of cola and describe its flavor. They were then told that if they chose to give up cola in favor of another option, they would not be able to drink the cola again and would never again enjoy the taste of cola. The second example was considered vital and involved the “*Loss of eyes.*” The participants were told that they would not be able to see for the rest of their lives if they chose this loss over another loss option. The participants' eyes were covered with a black cloth, and they were then asked to blindly search for a shuttlecock in the room. In this example, the participants were told that this task was neither a game nor an ability test but was being conducted so that they would experience the feeling of losing their eyes.

In Experiments 1 and 2, the participants underwent a loss decision making task after completing the loss examples. The participants were instructed to either press the left key if they chose to surrender the item presented on the left side, or to press the right key for the alternative option on the right side. They were informed that as soon as a choice was made, the related item would be lost to them forever. Next, the paired items were presented until a key-press response was recorded (for example, by pressing “1” with the left index finger or “4” with the right index finger). After each decision was made, the participants rated both the unpleasantness of the loss and the choice difficulty in Experiment 1 on a 7-point scale that ranged from 1 (not at all unpleasant or difficult) to 7 (extremely unpleasant or difficult). The participants rated the importance of the loss and the choice difficulty in Experiment 2 on a 7-point scale that ranged from 1 (not at all important or difficult) to 7 (extremely important or difficult) by pressing the corresponding numeric key on the computer. In both Experiment 1 and Experiment 2, a fixation cross was presented for 2 seconds between the self-reported ratings and the subsequent trial. The order of the the TT and TV conditions was randomized.

In Experiment 3, the participants performed a loss task after completing the loss examples. Looking at a computer screen, the participants were asked to imagine a situation in which they lost each of the alternatives. After a 6 second duration, a red square randomly framed one of the two options. The participants were informed that the framed option would be lost to them forever, and they were then asked to rate the unpleasantness of this loss on a 7-point scale that ranged from 1 (not at all unpleasant) to 7 (extremely unpleasant) by pressing the corresponding numeric key on the computer keyboard. A fixation cross was presented for 2 seconds between the self-reported rating and the subsequent trial. The order of the TR and TV conditions was randomized.

## Results

**Figure 1:**
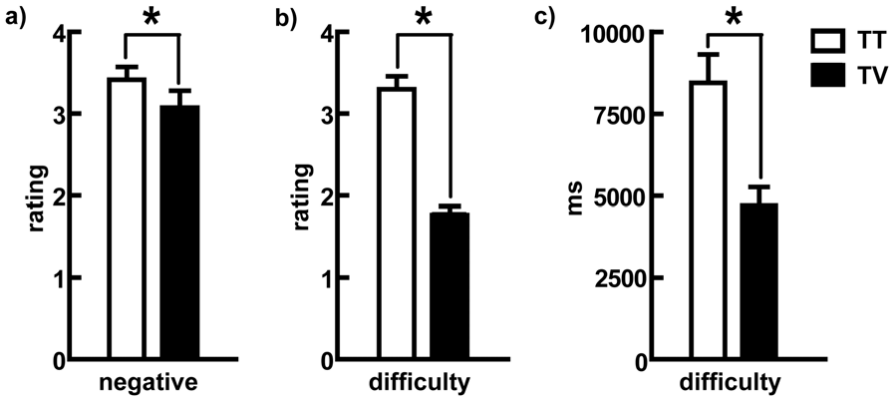
Self-Reported Negative Emotion Ratings and Response Times. a) Self-reported negative emotion ratings of choice in each loss decision condition. b) Self-reported difficulty ratings of choice in each loss decision condition. c) Mean response time (in milliseconds) in each loss decision condition.

The mean reaction times (RTs), self-reported difficulty and negative affect ratings of the loss decisions for Experiment 1 are shown in Figure 1 for two different conditions (TT and TV). Paired sample *t*-tests were conducted to test the differences between the average RTs (mean ± SE_TT_ = 8.45±.86; mean ± SE_TV_ = 4.69±.57 s), the self-reported difficulty of choice (mean ± SE_TT_ = 3.30±.16; M ± SE_TV_ = 1.76±.11), and the negative affect ratings of choice (mean ± SE_TT_ = 3.42±.16; mean ± SE_TV_ = 3.08±.21) under the two loss decision conditions. Compared with the participants in the TV condition, the participants in the TT condition felt more unpleasant, found the choices more difficult, and demonstrated longer RTs (*t*_negative_ = 2.20, *t*_difficulty_ = 8.45, *t*_RTs_ = 5.39, *ps*<.05). These results revealed that the contrast effects relieved negative emotions in the TV loss decision task (Figure 1). Additionally, to compare the differences of negative emotions between losing T in TT and TV, we performed a further analysis of covariance by controlling the difficulty with reaction time as a covariate. The results revealed a marginal significant main effect of condition, F(1,58) = 3.46, *p* = 0.07. The results indicated that the negative emotions induced by losing T in TT and TV decision were different, although we treated the difference in RTs between these two conditions as a covariate.

**Figure 2:**
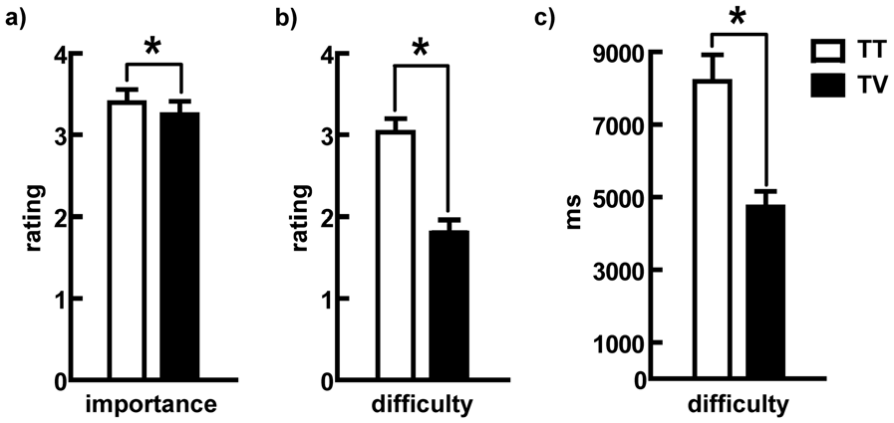
Self-Reported Importance Ratings and Response Times. a) Self-reported importance ratings for alternatives in each loss decision condition. b) Self-reported choice difficulty ratings in each loss decision condition. c) Mean response time (in milliseconds) in each loss decision condition.

For Experment 2, the mean reaction times (RTs), the self-reported choice difficulty and the importance ratings of the chosen item for two different conditions (TT and TV) are shown in Figure 2. Paired sample *t*-tests were conducted to test the differences between the RTs (mean ± SE_TT_ = 8.16±.73 s; mean ± SE_TV_ = 4.69±.43 s), the self-reported choice difficulty (mean ± SE_TT_ = 3.01±.17; M ± SE_TV_ = 1.78±.15), and the importance of the chosen items (mean ± SE_TT_ = 3.37±.16; mean ± SE_TV_ = 3.23±.16) under two loss decision conditions. Compared with losing T in TV, the participants in TT perceived T as being more important and more difficult to choose, and they spent a longer time deciding which choice to make (*t*_importance_ = 2.80, *t*_difficulty_ = 8.64, *t*_RTs_ = 8.16, *ps*<.01). These results revealed that the contrast of the alternatives changed the importance of the chosen options (Figure 2).

**Figure 3:**
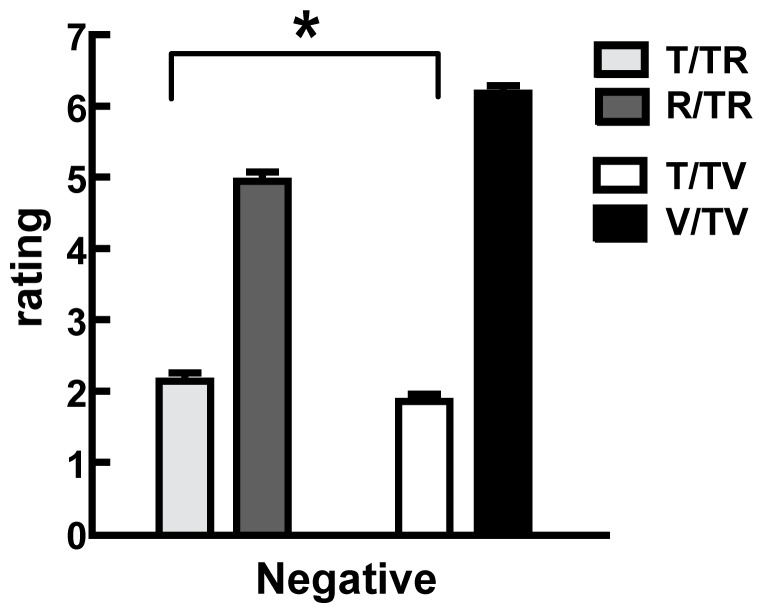
Self-Reported Negative Emotion Ratings. Self-reported negative emotion ratings of choice in each loss condition. Losing T in TV was rated as the least negative, followed by T in TR and R in TR, with V in TV rated as the most negative (*ps*<.01), even though the choice difficulty was controlled.

For Experiment 3, the mean self-reported negative affect ratings associated with imagining loss are shown for two different conditions (TR and TV) in Figure 3. Separate one-way repeated-measures analysis of variances (ANOVAs) were conducted to test the difference between the negative affect ratings in regards to loss under two conditions. The result revealed a significant main effect of conditions in the negative affect ratings of choice (F(3,116) = 491.12, *p*<.001). Furthermore, the Bonferroni correction indicated that the difference in the negative affect ratings of choice was significant (*ps*<.01): losing T in TV was rated as the least unpleasant (mean ± SE = 1.85±.11), followed by T in TR (mean ± SE = 2.14±.11) and R in TR (mean ± SE = 4.94±.13), with unpleasant ratings the highest for losing V in TV (mean ± SE = 6.18±.11). This result suggested that, compared to losing T in TR, the contrast effects relieved negative emotions when losing T in TV, even when the choice difficulty was controlled (Figure 3).

## Discussion

Although previous studies have demonstrated that contrast effects are ubiquitous in cognitive processing, very little research has examined whether emotions are affected by contrast effects in decision making. The present experiments revealed that contrast effects could moderate negative emotions.

### Contrast effects regulate the negative emotions in loss decisions

In study 1, we compared the difference between the negative emotion ratings in the VT and TT loss decisions. The results indicated that negative emotions might be affected by contrast effects.

Our current data revealed that compared with losing T in TV, participants felt more unpleasant when losing T in TT. Because both decision situations involved the loss of trivial items, we assumed that people would feel equal amounts of displeasure in both situations. Why were the self-reported negative emotions distinctly different between these conditions?

One possible explanation is that in TT and TV loss decisions, the negative emotions are affected by comparing the outcomes of both the chosen and unchosen options. Relative to the losing T, the vital item is the unchosen option in TV, while another ordinary item is the unchosen option in TT. When compared to protecting an item that is vital, losing an ordinary T appeared negligible in TV. In contrast, when compared to keeping another ordinary item, the loss of an ordinary T might be taken more seriously, which could lead to increased negative emotions. Some research has demonstrated that emotions depend on counterfactual comparisons of the outcomes of chosen versus unchosen options [29], [32]. For example, regret and disappointment are two types of emotions often associated with counterfactual comparisons of the outcomes of chosen and unchosen options. Regret is experienced when one's decision leads to an outcome that is a worse alternative [33], [34]; disappointment is experienced when the obtained outcome is worse than expected or hoped [35], [36]. An analysis of the emotional reactions of bronze and silver medalists at the 1992 Summer Olympics indicated that bronze medalists tended to be happier than silver medalists [16]. The authors attributed these results to the fact that the most compelling counterfactual alternative or unchosen option for the silver medalist was winning the gold, whereas for the bronze medalist, the most compelling counterfactul alternative or unchosen option was finishing without a medal. Thus, contrast effects, which are generated from the comparison of outcomes of chosen and unchosen options, regulate the negative emotions of TT and TV loss decisions [22].

In TT and TV loss decisions, the reference point changes the absolute loss into a relative loss, which leads to the variation of negative emotions. In their prospect theory, Kahneman and Tversky [37] argued that loss is a relative rather than an absolute concept. According to the reference point account, participants might think of losing trivial things (i.e., a less threatening outcome) as gains when compared to the possibility of losing vital things (i.e., a more threatening outcome). In other words, something particularly important can be protected [30], thus leaving decision makers with feelings of relief. Just as in the economic decision making process, consumers evaluate a monetary transaction against a reference. When a price is lower than one's reference point, people feel that they have “gained” relative to their reference point and vice versa [38]. In the prospect theory, a typical example of framing effects [39] was the Asian disease problem. This problem reflected a shift in the reference point because of different descriptions about the problem. The results indicated a general tendency for people to have a “gained” mentality when exposed to a gains (survival) format and a “lost” mentality when exposed to a losses (mortality) format [39]–[41]. As mentioned above, although losing T is equal in different decision situations, the loss of T that was changed into relative loss could be due to the distinct references; these references might cause the variation of negative emotions.

### The variations of importance aroused by contrast effects regulate negative emotions in loss decisions

In study 2, we compared the differences between the importance ratings of trivial options in TT and TV. This analysis was performed as a way to explore whether contrast effects could change the importance of trivial options differently in both conditions. In turn, the intensities of negative emotions would be differentially regulated.

Our current data revealed that, when compared with losing T in TV, participants perceived T as being more important in TT. Based on the results of study 2, how can we ascertain that the relative importance of trivial options were changed by contrast effects, which in turn regulated the intensities of negative emotions?

First, contrasts could modulate the level of subjective importance. Study 2 found that the importance ratings of the chosen T in TV were much lower than they were in TT because importance ratings are a type of subjective value that might change when compared between different items [8]–[13], [15]. Separate studies have provided evidence that subjective values changed depending on various comparisons, such as self-perceptions. For example, when exposed to photographs of attractive women with ideal physiques, women's perceptions of their own physical attractiveness, social physique anxiety, and social self-esteem were negatively affected. This is known as the physical attractiveness contrast effect, which involves a social comparison between the self and others [14]. Therefore, in the TT and TV loss decisions, the subjective value of the importance of the chosen T was modulated by the comparison with either a trivial T or a vital V.

Additionally, negative emotions might be affected by the importance of the loss. In the TT and TV loss decisions, the importance of the chosen T was differentially changed by the comparison with a trivial T or a vital V. Previous research has demonstrated that if the loss is more important to the decision maker, then elicited emotions will be more negative [42]–[47]. Hanselmann et al. [30] found that, when compared to losing an ordinary thing, losing a vital thing was perceived as much more emotionally distressing. Therefore, although there were equally trivial options lost in both the TT and TV loss decisions, the contrast effects might change the importance of trivial options differently in both conditions. Thus, the intensities of the negative emotions were differentially regulated.

### Even when controlling the impact of decision difficulty, contrast effects appear to regulate negative emotions

Previous research has demonstrated that negative emotions are affected by choice difficulty: the more difficult the trade-offs are, then the higher the elicited negative emotions [48]–[53]. In this regard, an alternative explanation concerning the lower negative emotions in TV than TT, which might be caused by an easy choice, deserves attention.

On the one hand, our fMRI study found that the observed relief-related emotion did not occur simply because the VT choices are easy. When we chose reaction times as an index of trade-off difficulty [51], the reward-related areas were not positively correlated with reaction time, whereas when we controlled for reaction times, the relief-related areas remained activated in VT compared to the TT loss decisions [22]. That is to say, excluding the decision difficulty, the contrast effects still regulate the activation in emotion-related areas.

On the other hand, in study 1, we compared the differences of negative emotions between losing T in TT and TV by controlling the difficulty (with reaction time as a covariate). The results suggested that the negative emotions induced by losing T in TV were lower than in the TT condition, although we controlled for the difficulty of choices. In study 3, the loss was predetermined, and the participants reported their negative emotion ratings for the loss. Compared to losing T in the TR loss condition, the results demonstrated that less negative emotions were elicited when losing T in the TV loss condition for different comparisons of R or V.

Moreover, we completed the following two control tasks (for details about the two tasks, please see *Supplementary Materials S1*). For Control task 1, the materials were the same as in study 2. The difference between the two tasks was that in Control task 1, after choosing the unimportant item from every pair of nouns, the participants were asked to rate their negative emotional state at once. The results indicated that choice difficulty was higher in the TT condition than than it was in the TV condition, but there were no difference between negative emotions in the TT and TV conditions. These results revealed that in the non-emotive decision task, the negative emotions could be the same although the choice difficulties were different. Previous research [48]–[53] have found that, in the studies of motivational conflict, the more difficult the trade-offs are, the higher the elicited negative emotions are, and negative emotions could be reduced by choice difficulty. However, our control task suggested that decision difficulty did not necessarily lead to the relief of negative emotions in the non-emotive decision task. For Control task 2, the materials were the same as study 3. The only difference between the two tasks was that, in Control task 2, the participants were forced to surrender one item of every pair of nouns, and then rate their negative emotion when losing the item at once. The results suggested that participants in the TR condition felt more unpleasant than the participants in the TV condition, but there were no difference between the choice difficulties in the TR and TV conditions. These results revealed that the contrast effects could moderate the negative emotions, although the choice difficulties were the same.

The above evidence led us to believe that contrast effects can still affect the loss-related negative emotions, even if we exclude the impact of choice difficulty.

Although the current study employed hypothetical decision problems, the converging evidence has shown that emotion can be produced and studied in a hypothetical manner [22], [30], [54]–[59]. Thus, in our study, hypothetical situations in our studies can be effective in eliciting emotions during decision making.

### Limitations and Prospects

Although we expect that contrast effects can bring about any long-lasting reduction/changes in negative emotions, and that PANAS are capable of measuring longer-term emotional states [60], too many items in the mood questionnaire might increase the subjects' fatigue and aversion. This problem lowers the credibility and sensitivity of the assessment results [30], if the participants have to perform the emotional assessment after every decision making task. At the same time, a single item assessment of subjective emotion might be unable to evaluate the subjects' long-term emotional state. Thus, our current research focuses on the immediate variations of negative emotions. Further research might be needed to explore the long-term sustained effect of contrast effects regulation of emotional states.

Our current study found that contrast effects could regulate emotions. This is, to our knowledge, the first study to demonstrate that contrast effects can alleviate a negative affect in loss-related decision making. These findings will enrich and develop the literature on emotion regulation theory.

Contrast effects are a cost-effective mitigation strategy for negative emotions. Previous research suggests that social support [61] and money [61], [62] are common ways to alleviate human negative emotions. However, these methods cost enormous human and financial resources, and they sometimes result in little effect. For instance, once a person has begun using money to reduce his distress, the likelihood of selfish, asocial behavior will increase [63], [64], which might result in a vicious cycle that damages social relationships [65]. However, contrast effects could mitigate the negative emotions only when comparing the loss of equally important options in different situations [22], [30]. The movie Sophie's Choice presents a vivid example of how contrast effects mitigate negative emotions. Sophie felt sad when she was forced to accept her own rape. However, when she had to face either her own rape or the loss of her son, she chose to lose her chastity, but she smiled for saving her son's life [22]. Our present and previous research [22] have found that, participants felt very unhappy when forced to lose either jewelry or a car, however, their negative emotions were relieved whent they were forced to lose either legs or a car, although the same car was lost in both situations. Thus, contrast effects can moderate negative emotions in a more cost-effective way.

## Supporting Information

Supplementary Materials S1 Supplemental Data.
(DOC)
