# Supplementary material for: Can Contrast Effects Regulate Emotions? A Follow-Up Study of Vital Loss Decisions
Source: PLoS One. 2012 Aug 8;7(8):e42763. doi: 10.1371/journal.pone.0042763 (PMC3414460; doi:10.1371/journal.pone.0042763)
Supplement: Supplementary Materials S1 — Supplemental Data. (DOC) [file pone.0042763.s001.doc]

**Supplemental Data**

**Can Contrast Effects Regulate Emotions? A Follow-up Study of Vital Loss Decisions**

Qi Li, Yue Qi, Xianyun Liu, Jing Luo

**This Word file includes:** Methods and Results for two control tasks

**Materials and Methods**

Participants

Nineteen healthy university students participated in Control task 1 (7 males, 23.74 ± .60 years old) and seventeen students in Control task 2 (6 males, 22.47 ± .49 years old). All of the participants reported a lack of neurological or psychiatric history. Each participant voluntarily enrolled and signed an informed consent statement prior to the experiment. The procedure was approved by the Institutional Review Board of the Institute of Psychology, Chinese Academy of Sciences.

Stimuli

For Control task 1, the materials were the same as the materials in Experiment 2. For Control task 2, the materials were the same as the materials in Experiment 3.

Procedure

In Control task 1, the participants were instructed to either press the left key if they thought the item presented on the left side was unimportant or to press the right key for the alternative option on the right side. Next, the paired items were presented until a key-press response was recorded (for example, by pressing “1” with the left index finger or “4” with the right index finger). After each decision was made, the participants rated the negative affect ratings on a 7-point scale that ranged from 1 (not at all unpleasant) to 7 (extremely unpleasant) by pressing the corresponding numeric key on the computer. A fixation cross was presented for 2 seconds between the self-reported ratings and the subsequent trial. The order of the TT and TV conditions was randomized.

In Control task 2, prior to the test, participants completed two specific “loss” examples as a way to maximally involve them in the experimental situation. The two examples were the same as the examples in Experiment 1 and 3.

The participants underwent a loss decision making task after completing the loss examples. The participants were instructed to either press the left key if they chose to surrender the item presented on the left sideor to press the right key for the alternative option on the right side. They were informed that as soon as a choice was made, the related item would be lost to them forever. Next, the paired items were presented until a key-press response was recorded (for example, by pressing “1” with the left index finger or “4” with the right index finger). After each decision was made, participants rated the negative affect ratings of the loss on a 7-point scale that ranged from 1 (not at all unpleasant) to 7 (extremely unpleasant) by pressing the corresponding numeric key on the computer. A fixation cross was presented for 2 seconds between the self-reported ratings and the subsequent trial. The order of the TR and TV conditions was randomized.

**Results**

The mean reaction times (RTs) and negative affect ratings of the loss decisions for Control task 1 were analyzed for two different conditions (TT and TV). Paired sample *t*-tests were conducted to test the differences between the average RTs (mean ± SE_TT_ = 3.61 ± .21; mean ± SE_TV_ = 2.32 ± .13s), and the negative affect ratings of choice (mean ± SE_TT_ = 1.83 ± .17; mean ± SE_TV_ = 1.66 ± .13) under the two loss decision conditions. Compared with the participants in the TV condition, the participants in the TT condition found the choices more difficult. These participants demonstrated longer RTs (*t*_RTs_ = 8.27, *p* < .001) but no difference between negative emotions (*t*_negative_ = 1.82 , *p >* .05). These results revealed that in the non-emotive decision task, the negative emotions could be the same even if the difficulty of the decisions was different.

For Control task 2, the mean reaction times (RTs), and negative affect ratings for the loss decisions were analyzed for two different conditions (TV and TR). Paired sample *t*-tests were conducted to test the differences between the RTs (mean ± SE_TV_ = 4.18 ± .81; mean ± SE_TR_ = 4.91 ± .50s) and the negative affect ratings of choice (mean ± SE_TV_ = 2.56 ± .85; mean ± SE_TR_ = 2.77 ± .87) under two loss decision conditions. Compared with those in the TV condition, the participants in the TR condition felt more unpleasant (*t*_negative_ = 2.25, *p* < .05), but there was no difference between the RTs (*t*_RTs_ = 1.65, *p* >.05). These results revealed that the contrast effects could moderate the negative emotions in the TV loss decision task, although the difficulties of the two decision conditions were the same.
